# The YABBY Transcription Factor, SlYABBY2a, Positively Regulates Fruit Septum Development and Ripening in Tomatoes

**DOI:** 10.3390/ijms25105206

**Published:** 2024-05-10

**Authors:** Hui Shen, Baobing Luo, Yingfeng Ding, Haojun Xiao, Guoping Chen, Zhengan Yang, Zongli Hu, Ting Wu

**Affiliations:** 1 Laboratory of Molecular Biology of Tomato, Bioengineering College, Chongqing University, Chongqing 400030, China; hbshenhui@163.com (H.S.); baobingluo@163.com (B.L.); chenguoping@cqu.edu.cn (G.C.); 2 Key Laboratory of Vegetable Biology of Yunnan Province, College of Landscape and Horticulture, Yunnan Agricultural University, Kunming 650201, China; 18313024561@163.com (Y.D.); 18996446327@163.com (H.X.); yangzhengan@ynau.edu.cn (Z.Y.)

**Keywords:** YABBY, SlYABBY2a, tomato, septum, auxin, ripening

## Abstract

The tomato fruit is a complex organ and is composed of various structures from the inside out, such as columella, septum, and placenta. However, our understanding of the development and function of these internal structures remains limited. In this study, we identified a plant-specific YABBY protein, SlYABBY2a, in the tomato (*Solanum lycopersicum*). *SlYABBY2a* exhibits relatively high expression levels among the nine YABBY genes in tomatoes and shows specific expression in the septum of the fruit. Through the use of a gene-editing technique performed by CRISPR/Cas9, we noticed defects in septum development in the *Slyabby2a* mutant fruits, leading to the inward concavity of the fruit pericarp and delayed septum ripening. Notably, the expression levels of key genes involved in auxin (*SlFZY4*, *SlFZY5*, and *SlFZY6*) and ethylene (*SlACS2*) biosynthesis were significantly downregulated in the septum of the *Slalkbh10b* mutants. Furthermore, the promoter activity of *SlYABBY2a* was regulated by the ripening regulator, SlTAGL1, in vivo. In summary, these discoveries provide insights into the positive regulation of SlYABBY2a on septum development and ripening and furnish evidence of the coordinated regulation of the auxin and ethylene signaling pathways in the ripening process, which expands our comprehension of septum development in the internal structure of the fruit.

## 1. Introduction

The tomato is considered the most valuable horticultural commodity worldwide and is a significant source of micronutrients in the human diet [1]. From their wild progenitors to modern cultivated varieties, artificial selection has amplified tomato fruit weight by approximately 50-fold, concomitant with profound alterations in fruit metabolism [2,3]. In the context of commercially cultivated tomatoes, fruit morphology and ripening characteristics stand as pivotal traits. For instance, the *fs8.1* locus, associated with tomato fruit shape variation from round to elongated (oblong or square), represents a critical differentiating factor between fresh consumption and processing tomatoes [4]. However, our comprehension the internal structure development of tomatoes and its influences on fruit morphology and quality remains insufficient.

Fruit development initiates from the fruit set, and the resumption of cell division within the ovary signifies a successful fruit set [5,6]. After the fruit set, early tomato fruit development is often described as two consecutive stages. The first stage involves cell division, followed by cell expansion [7,8]. The levels of gene expression related to the cell cycle regulate the process of cell division. The continuous process of mitotic cell division is regulated by a heterodimeric protein complex composed of cyclin-dependent kinase (CDK) catalytic subunits and regulatory subunits (CYC) [9]. Auxin is also involved in regulating cell division. For instance, in pre-anthesis ovaries of *Arabidopsis*, Aux/IAA interferes with auxin signaling by binding to the auxin response factor ARF, thereby inhibiting cell division and maintaining the ovary in a dormant state [10]. The increase in auxin levels due to the completion of pollination in flowers leads to the degradation of Aux/IAA and the release of ARF, which then activates the expression of target genes to promote cell division. [11]. Additionally, auxin has the ability to control the expression of auxin-responsive genes and influence cell expansion through the action of inhibitory proteins, like IAA17. In tomatoes, *IAA17* exhibits high expression levels at the onset of fruit cell expansion (10 DPA—days post anthesis), gradually decreasing until fruit ripening [12]. Reducing the expression of the *IAA17* through RNAi resulted in enlarged fruit pericarp cells, ultimately leading to the development of larger fruits [8]. Fruit development directly impacts its size and shape. Mutants such as *sun*, *ovate*, *lc*, and *fs8.1* exhibit significant changes in tomato fruit shape, making them essential genetic materials for studying fruit morphology [13]. Among them, the mutated *sun* variant exhibits heightened cell numbers along the proximal–distal axis, resulting in elongation. Alterations in the expression of genes associated with auxin production, signaling, and transport indicate that SUN may influence fruit shape during ovary development by modulating auxin-related gene expression [13].

Fruit ripening constitutes the last phase of fruit development, with auxin playing a role in controlling the shift from unripe to ripe fruit. In tomatoes, auxin signaling sharply decreases during wild-type (WT) fruit ripening, but this decrease is absent in the *ripening inhibitor* (*rin*) mutant [14]. Additionally, the upregulation of *SlSAUR69* expression led to early ripening, while the downregulation of *SlSAUR69* prolonged the ripening process [14]. The use of chromatin immunoprecipitation (ChIP)-ChIP, ChIP-seq, and RNA sequencing in combinatorial analysis indicated that *SlSAUR69* is a direct target of RIN [15,16], a SEPALLATA (SEP) clade protein. This implies that RIN could play a role in regulating auxin during fruit ripening. Notably, RIN is particularly known for forming DNA-binding complexes with the MADS transcription factors TAGL1, FUL1, and FUL2, which play a role in regulating ripening [17,18,19,20,21,22,23,24]. One example is that RIN, FULs, and TAGL1 have the ability to directly attach to the *SlACS2* gene promoter [15,17,20], which controls the production of ethylene, a key regulator in tomato fruit ripening. However, whether TAGL1 and FULs affect the auxin signaling pathway and fruit ripening has seldom been reported. Early studies have shown that the *TAGL1* gene regulates fruit development and ripening. A reduction in TAGL1 mRNA in tomato plants results in yellow-orange fruits with lower carotenoid levels and thinner pericarps [18]. Moreover, the methylated form of *TAGL1*, known as the *GREEN STRIPE* (*GS*) locus, affects various aspects of chloroplast development and carotenoid buildup [25]. Furthermore, silencing *SlTAGL1* through RNAi technology in Ailsa Craig (AC) or knocking out *SlTAGL1* using CRISPR/Cas9 in the *gs* both resulted in the formation of sunken fruit phenotypes [18,25]. However, the mechanism by which SlTAGL1 influences this fruit shape formation remains unknown.

The naming of the YABBY transcription factor (TF) family originated from studies in *Arabidopsis thaliana*. The *crc-1* mutant of *Arabidopsis* exhibited incomplete fusion of the carpel margins at the apex, resembling the shape of a crab’s claw. As a result, this gene was named *CRC* (*CRABS CLAW*) [26]. The *CRC* gene encodes a specific TF with a zinc finger domain and a helix–loop–helix domain. By utilizing this structural characteristic, five additional genes were discovered in the *Arabidopsis* genome, all showing high similarity in their zinc finger and helix–loop–helix domains. This led to the definition of a new gene family. To establish a connection with the founding member CRC, Bowman and Smyth named this gene family after the Australian freshwater crayfish “yabby”, giving rise to the unique YABBY TF family in plants [26]. Currently, YABBY proteins have been identified in numerous species, with six in *Arabidopsis thaliana* [27], nine in *Oryza sativa* (rice) [28], nine in *Solanum lycopersicum* (tomato) [29], and seven in *Vitis vinifera* (grape) [30]. Based on the evolutionary relationships and functional studies in *Arabidopsis*, YABBY proteins in dicotyledonous plants such as *Arabidopsis* and tomato can be categorized into five subfamilies: FIL/YAB3, CRC, INO, YAB2, and YAB5 [29,31]. The main characteristic of YABBY proteins is their role in the abaxial cells of lateral organs in plants [32]. Furthermore, recent research has demonstrated that YABBY family genes play a crucial role in various aspects of plant reproductive growth [33,34], vegetative growth [35], and response to abiotic stresses [36].

The natural mutations of *fasciated* (*fas*) and *locule number* (*lc*) during the domestication of tomato fruits had a combined impact on the size of the meristematic tissue and the number of locules in tomato fruits [37,38]. Initially, the putative gene for *fas* was identified as *SlYABBY2b* [39]. Subsequent studies revealed that an inversion of 296 kb at the *fas* locus affected the promoter of *SlCLV3*, thereby altering the expression level of *SlCLV3* [40,41,42]. Although to a lesser extent than *SlCLV3*, *SlYABBY2b* also participates in regulating locule number. The overexpression of *SlYABBY2b* decreased the number of locules in fruits [43], while the knockout of *SlYABBY2b* led to dwarfed plants with smaller flowers and fruits [44]. Among other members of the tomato YABBY family, SlCRCa acts as an inhibitor of flower organ size and negatively impacts fruit size by influencing cell division and expansion [33]. Furthermore, in single-knockout mutants of *SlCRCa* or *SlCRCb*, uncertain flower development was observed, while in double-mutant lines of *SlCRCa* and *SlCRCb*, all flowers exhibited severe phenotypic uncertainty, indicating that tomato CRC homologous genes ensure the normal initiation of floral meristem and carpel development through partial redundancy [34]. Altogether, these studies suggest that the tomato YABBY family plays a role in controlling the development of floral organs and fruits. However, the tomato YABBY family consists of nine shared members, most of which have unknown functions.

The tomato fruit consists of various structures, such as the placenta, seeds, locular tissue, septum, and total pericarp, from the inside out [45]. Our understanding of how these structures influence fruit development and ripening still needs to be improved. A thorough examination of the tomato fruit transcriptome, covering various tissues and development stages, has provided insights into gene expression patterns in different periods and structures of tomato fruit [45]. Furthermore, the AGAMOUS (AG) MADS-box TF SlMBP3 has been identified as a key regulator of locular tissue in tomato fruit, impacting fruit softening [46,47]. These studies have begun to focus on the developmental regulation of internal structures within tomato fruit. The septum in tomato fruit serves as a specialized tissue that separates different locules and may have a structural function. However, the developmental regulation of the septum remains largely unexplored. This research uncovered that *SlYABBY2a* serves as a specific regulator of septum development and ripening. Knocking out *SlYABBY2a* resulted in altered expression of genes related to auxin and ethylene signaling pathways in the septum, ultimately leading to inward collapse at the septum and delayed septum ripening. In addition, the MADS-box TF SlTAGL1 can activate the promoter of *SlYABBY2* in vivo, indicating that *SlYABBY2* is a direct target of SlTAGL1. These findings offer fresh insights into the function of YABBY TF in septum development and ripening.

## 2. Results

### 2.1. Characterization of the Transcription Factor SlYABBY2a

**Figure 1:**
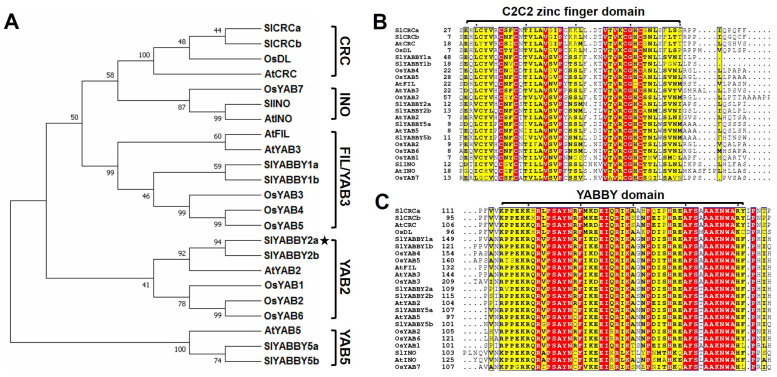
Phylogenetic relationship and conservation analysis of YABBY family proteins. (**A**) Phylogenetic tree. The asterisk is used to highlight the location of SlYABBY2a. (**B**,**C**) Multiple sequence alignments of conserved C2C2 zinc finger (**B**) and YABBY domains (**C**). Sl: *Solanum lycopersicum* (tomato); At: *Arabidopsis thaliana*; Os: *Oryza sativa* (rice). Red represents highly conserved amino acids in the sequence alignment, yellow represents partially conserved, and dots represent gaps.

**Figure 2:**
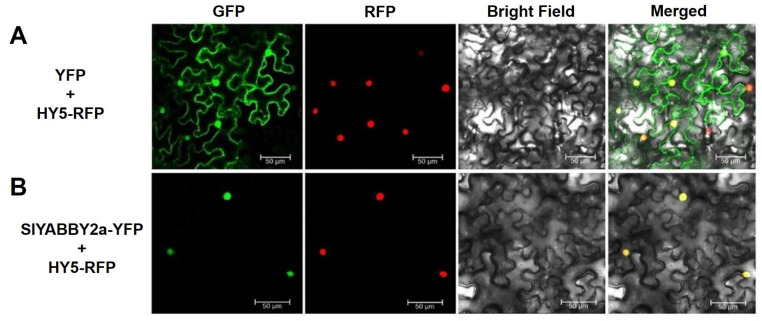
Subcellular localization of SlYABBY2a. (**A**) The green fluorescent signal excited by YFP was observed in both the nucleus and cytoplasm. (**B**) The SlYABBY2a-YFP fusion protein produced a green fluorescent signal in the nucleus upon excitation. The HY5-RFP protein served as a positive control for nuclear localization. The red fluorescence signal is emitted by RFP (red fluorescent protein), while the green fluorescence signal is emitted by YFP (yellow fluorescent protein). The yellow signal is formed by the convergence of red and green fluorescence signals. The circles under the bright field provide the outline of the cells. Scale bar = 50 µm.

In order to study the evolutionary connection of SlYABBY2a in tomatoes (*Solanum lyco-persicum*), a phylogenetic tree was created using the complete amino acid sequences of YABBY proteins from model plants, like tomatoes, *Arabidopsis*, and rice. According to the YABBY family protein classification and naming system in *Arabidopsis*, the tomato YABBY family proteins were categorized into five distinct subfamilies: YAB2, YAB5, FIL/YAB3, INO, and CRC (Figure 1A). Notably, there is no homologous protein of YAB5 in the rice genome, which is a monocotyledonous plant. In addition, tomato and *Arabidopsis* are dicotyledonous plants. Still, the tomato has two proteins in the YAB2, YAB5, and CRC subfamilies, while *Arabidopsis* has only one protein, suggesting potential functional differentiation of tomato proteins, respectively, within the YAB2, YAB5, and CRC subfamily. Further amino acid sequence analysis demonstrated that SlYABBY2a shares a C2C2 zinc finger domain (Figure 1B) and a YABBY domain (Figure 1C) with other YABBY proteins. In addition, we carried out transient expression assays through agroinfiltration to observe the subcellular distribution of YFP and recombinant proteins. The control consisted of an empty vector, and we detected green fluorescent signals in the nucleus and cytoplasm (Figure 2A). Conversely, the green fluorescent signals emitted by the SlYABBY2a-YFP fusion protein overlapped with the nucleus localization red fluorescence signal emitted by the HY5-YFP fusion protein (Figure 2B). These findings corroborated that SlYABBY2a is a conserved YABBY family transcription factor and localizes to the nucleus.

### 2.2. SlYABBY2a Is Specifically Expressed in the Septum of Tomato Fruit

**Figure 3:**
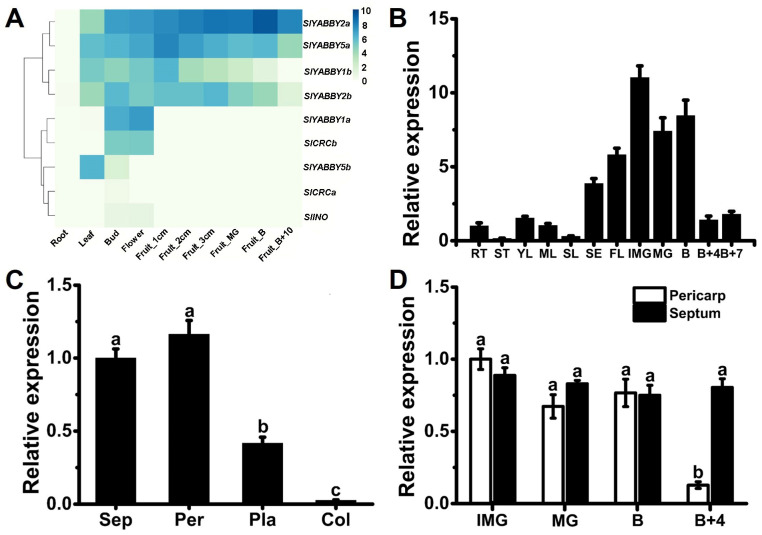
Expression analyses of *SlYABBY2a*. (**A**) Gene expression heatmap of the tomato YABBY family genes based on transcriptome expression data (FPKM) taken from the Tomato Genome Consortium, 2012 [48]. Each column represents a different tomato tissue or organ, including different fruit developmental stages. (**B**) Tissue- and organ-specific relative expression pattern of *SlYABBY2a* detected by qRT-PCR. RT, root; ST, stem; YL, young leaf; ML, mature leaf; SL, senescent leaf; SE, sepal; FL, flower; IMG, immature green; MG, mature green; B, breaker stage; B + 4, four days after breaker stage; B + 7, seven days after breaker stage. (**C**) Relative expression levels of *SlYABBY2a* in different fruit tissues at the B stage. Sep, septum; Per, pericarp; Pla, placenta; Col, columella. (**D**) Relative expression levels of *SlYABBY2a* in pericarp and septum at different stages of fruit ripening. Each value represents the mean ± SE of three biological replicates. The same lowercase letters indicate no significant difference using one-way ANOVA and Duncan’s multiple range test (*p* < 0.05).

To investigate the gene expression levels of nine YABBY TF family genes in tomato plants, we conducted a comprehensive analysis using transcriptome data (Tomato Genome Consortium, 2012 [48]). The YABBY family genes in tomatoes exhibited minimal expression in the roots, with some genes displaying tissue-specific expression patterns (Figure 3A). For instance, higher expression levels of *SlYABBY5b* were observed in the leaf and bud compared to other tissues, while *SlYABBY1a* and *SlCRCb* showed elevated expression levels in the bud and flower. Additionally, four genes within the YABBY family, *SlYABBY2a*, *SlYABBY5a*, *SlYABBY1b*, and *SlYABBY2b*, exhibited more widespread expression patterns. Notably, *SlYABBY2a* displayed relatively high expression levels in all tissues, except the roots (Figure 3A). Furthermore, the expression pattern of *SlYABBY2a* in tomato tissues or organs was investigated through qRT-PCR, revealing that *SlYABBY2a* has relatively high expression levels in flowers and fruits (up to the breaker stage) (Figure 3B). Interestingly, in another dataset of spatial and temporal transcriptome data during tomato fruit development, we found that *SlYABBY2a* exhibited abundant expression in the pericarp and septum, while its expression levels were significantly lower in structures, such as the placenta and columella (Figure S1). To validate this tissue-specific expression pattern of *SlYABBY2a*, we performed qRT-PCR to assess its expression levels in different fruit tissues at the Breaker stage. The findings demonstrated that the relative expression levels of *SlYABBY2a* were significantly elevated in the pericarp and septum compared to the placenta and columella (Figure 3C). The expression levels of *SlYABBY2a* were further compared in the pericarp and septum of fruits at different developmental stages. The results showed that during fruit development stages, including immature green (IMG), mature green (MG), and breaker (B), there were no significant differences observed in the expression levels of *SlYABBY2a* between the pericarp and septum. However, during ripening, specifically at the B + 4 stage, the expression level of *SlYABBY2a* significantly decreased in the pericarp, while it remained similar to the IMG stage in the septum (Figure 3D). These results suggested that *SlYABBY2a* is expressed explicitly in the septum of fruits, indicating its potential role in controlling the development and ripening process of fruit septum.

### 2.3. Knocking out of SlYABBY2a Affects the Development of Fruit Septum

**Figure 4:**
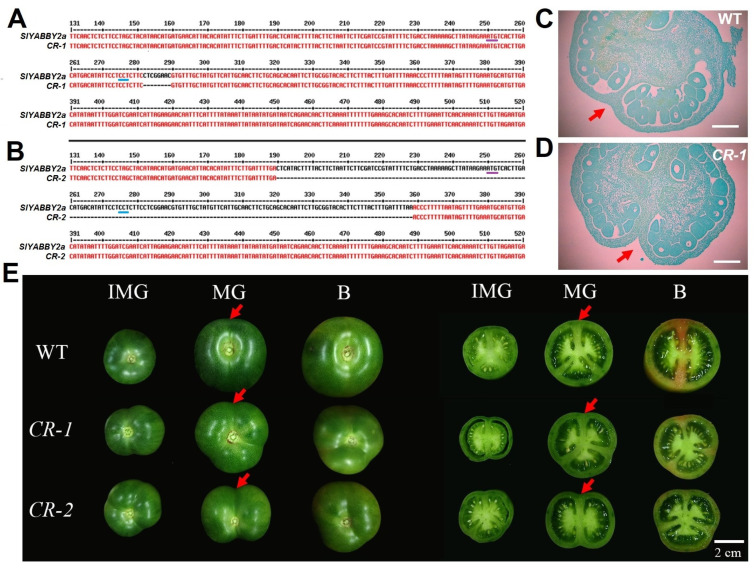
Phenotypic characterization of the *Slyabby2a* mutant during fruit development. (**A**,**B**) The genotypes of *Slyabby2a* mutant lines *CR-1* (**A**) and *CR-2* (**B**). The short purple and blue underlines indicate the initiation codon and PAM sequence “CCT”, respectively. (**C**,**D**) Carpel section of WT (**C**) and *CR-1* (**D**) one day after flowering. WT, wild type. The red arrows indicate the carpel’s fusion and the septum’s formation. Scale bar = 300 µm. (**E**) Fruit phenotype of WT, *CR-1,* and *CR-2* at the different fruit development stages. IMG, immature green; MG, mature green; B, breaker stage. Red arrows indicate the connection position of the septum and pericarp.

To further investigate the biological functions of *SlYABBY2a*, we successfully obtained two homozygous mutant lines of *SlYABBY2a* with distinct genotypes, *CR-1* and *CR-2*, through gene editing techniques, genetic transformation, and genome sequencing (Figure S2). Specifically, *CR-1* exhibited an 8 bp deletion near the initiation codon (Figure 4A), while *CR-2* exhibited a 169 bp base deletion, including the initiation codon of *SlYABBY2a* (Figure 4B). These deletions induced a frameshift mutation in the coding sequence, resulting in premature translation termination. Gene expression analysis of *SlYABBY2a* revealed its specific expression in the fruit pericarp and septum. In addition, the septum was formed by the fusion of carpels. Therefore, we conducted paraffin sectioning and observation of the carpel tissue from WT and *Slyabby2a* mutants one day after flowering. Compared to the WT, the cell layers at the junction of carpels in the *Slyabby2a* mutants were noticeably separated, and the degree of carpel fusion was reduced (Figure 4C,D), confirming the involvement of *SlYABBY2a* in carpel fusion and septum development. Moreover, during the further fruit development stages, the fruit of *Slyabby2a* mutants exhibited an uneven fruit appearance, and significant invagination at the junction of the fruit septum and pericarp was observed (Figure 4E). Hence, these findings confirmed the biological function of *SlYABBY2a* in controlling septum development and influencing fruit shape.

### 2.4. Knocking out of SlYABBY2a Alters the Auxin Signaling Pathway in the Fruit Septum

**Figure 5:**
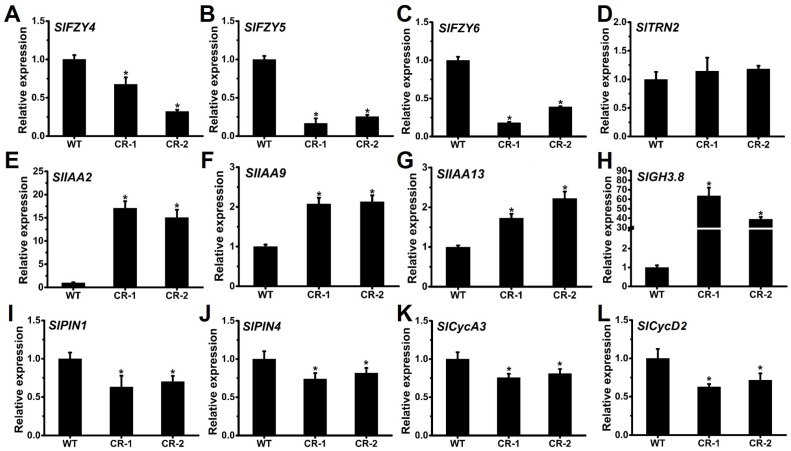
Transcript quantification of fruit septum at 20 days post anthesis stage from WT and *Slyabby2a* lines. (**A**–**C**) The relative expression of auxin synthesis genes *SlFZY4* (**A**), *SlFZY5* (**B**), and *SlFZY6* (**C**). (**D**–**H**) The relative expression of auxin response inhibitor genes *SlTRN2* (**D**), *SlIAA2* (**E**), *SlIAA9* (**F**), *SlIAA13* (**G**), and *SlGH3.8* (**H**). (**I**,**J**) The relative expression of auxin transport genes *SlPIN1* (**I**) and *SlPIN4* (**J**). (**K**,**L**) The relative expression of cell cycle-related genes *SlCycA3* (**K**) and *SlCycD2* (**L**). Each value represents the mean ± SE of three biological replicates. Asterisks indicate significant differences between WT and each *Slyabby2a* mutant line, CR-1 and CR-2 (* *p* < 0.05; Student’s *t*-test).

Auxin plays a crucial role in regulating tomato fruit development, with its main signaling pathway consisting of three stages: synthesis, signal transduction, and response [49]. YABBY family transcription factors, such as AtCRC, have been reported to directly participate in regulating auxin homeostasis in the carpel primordia [50,51]. To further investigate the pericarp invagination phenotype observed in *Slyabbay2a* mutants, the expression levels of known auxin-related genes were compared between the fruit septum of WT and *Slyabbay2a*. Three auxin synthesis genes (*SlFZY4*, *SlFZY5*, and *SlFZY6*) [52], five auxin response inhibitor genes (*SlIAA2*, *SlIAA9*, *SlIAA13*, *SlGH3.8*, and *SlTRN2*) [44,53], and two auxin transport genes (*SlPIN1* and *SlPIN4*) [54] were selected (Figure 5). Quantitative RT-PCR analysis showed a significant decrease in the transcripts of auxin synthesis genes (*SlFZY4*, *SlFZY5*, and *SlFZY6*; Figure 5A–C), as well as the auxin transport genes (*SlPIN1* and *SlPIN4*; Figure 5I,J), in the fruit septum of *Slyabbay2a* mutants compared to the WT. However, the expression levels of the five tested auxin response inhibitor genes were significantly higher in the fruit septum of *Slyabbay2a* mutants compared to the WT (Figure 5E–H). Furthermore, since auxin can regulate the synthesis of cyclin-dependent kinase (CDK) complexes through the regulation of auxin response genes (ARGs), thereby affecting the G1/S transition in plants [55], we further examined the transcript levels of two cell cycle-related genes. The results showed that the transcript levels of cell cycle-related genes, *SlCycA3* [56] and *SlCycD2* [57], were also significantly downregulated in the fruit septum of *Slyabbay2a* mutants (Figure 5K,L). These findings suggested that the auxin signaling pathway is altered in the fruit septum due to the knockout of *SlYABBY2a*, leading to changes in fruit development.

### 2.5. Knocking out SlYABBY2a Affects the Ripening of Fruit Septum

**Figure 6:**
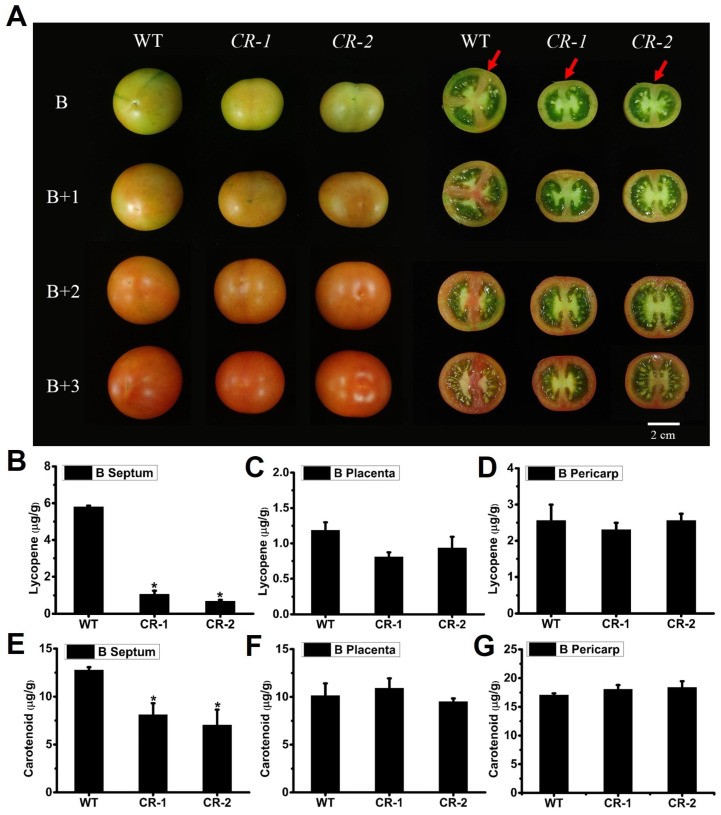
Phenotypic characterization of the *Slyabby2a* mutant during fruit ripening. (**A**) Fruit phenotype of WT, *CR-1,* and *CR-2* at different fruit ripening stages. B, breaker stage; B + 1, one day after breaker stage; B + 2, two days after breaker stage; B + 3, three days after breaker stage. Red arrows indicate the connection position of the septum and pericarp. Scale bar = 2 cm. (**B**–**D**) Lycopene contents in the septum (**B**), placenta (**C**), and pericarp (**D**) from WT and *Slyabby2a* mutant fruits at the B stage. (**E**–**G**) Total carotenoid contents in the septum (**E**), placenta (**F**), and pericarp (**G**) from WT and *Slyabby2a* mutant fruits at the B stage. Red arrows indicate the connection position of the septum and pericarp. Error bars indicate the SE based on three replicates. Asterisks indicate significant differences between WT and each *Slyabby2a* mutant line, CR-1 and CR-2 (* *p* < 0.05; Student’s *t*-test).

During our observation of the fruit shape and cross-section of *Slyabbay2a* mutants, we noticed that the maturity of the *Slyabbay2a* fruit septum in the breaker (B) stage was significantly lower compared to the WT. For instance, the septum of WT fruit had turned red, indicating the accumulation of lycopene, while the septum of *Slyabbay2a* mutants remained green, resembling an unripened state (Figure 4E). This finding prompted us to investigate whether *SlYABBY2a* plays a role in controlling the ripening of the fruit septum. To test this hypothesis, we further observed fruits at different stages of ripening. Throughout the ripening process of the fruit, no significant variations were observed in the external fruit color or pigment accumulation between *Slyabbay2a* mutants and the WT (Figure 6A). However, the cross-sectional analysis demonstrated a rapid increase in pigment accumulation in the septum of WT fruits as they matured, while *Slyabbay2a* mutant fruits experienced a significant delay in pigment accumulation (Figure 6A). The ripeness of tomatoes is typically determined by the levels of total carotenoids and lycopene. Therefore, we sampled the fruit pericarp, septum, and placenta tissues from WT and *Slyabbay2a* mutant fruits and measured the levels of total carotenoids and lycopene. The findings revealed a significant decrease in the levels of total carotenoids and lycopene in the fruit septum of *Slyabbay2a* mutants compared to WT fruits (Figure 6B,C). At the same time, there were no differences in the levels of total carotenoids and lycopene in the fruit pericarp and placenta tissues (Figure 6C,D and Figure 6F,G). These findings indicated that the knockout of *SlYABBY2a* specifically impacts the ripening process of the fruit septum.

### 2.6. Knocking out SlYABBY2a Alters Ripening-Related Genes in the Fruit Septum

**Figure 7:**
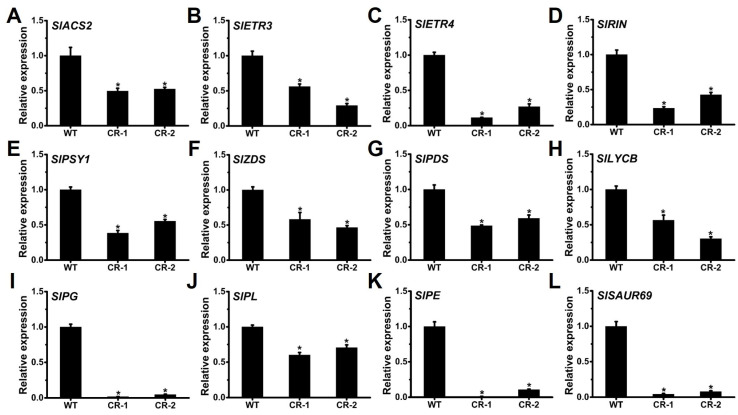
Transcripts quantification of the fruit septum at the B stage from WT and *Slyabby2a* lines. (**A**–**C**) Relative expression of ethylene biosynthesis and receptor genes *SlACS2* (**A**), *SlETR3* (**B**), and *SlETR4* (**C**). (**D**) Relative expression of the master regulator gene *SlRIN* of tomato fruit ripening. (**E**–**H**) Relative expression of carotenoid biosynthesis genes *SlPSY1* (**E**), *SlZDS* (**F**), *SlPDS* (**G**), and *SlLYCB* (**H**). (**I**–**K**) Relative expression of cell wall-modifying genes *SlPG* (**I**), *SlPL* (**J**), and *SlPE* (**K**). (**L**) Relative expression of the auxin transport repressor gene *SlSAUR69*. Error bars indicate the SE based on three replicates. Asterisks indicate significant differences between WT and each *Slyabby2a* mutant line, CR-1 and CR-2 (* *p* < 0.05; Student’s *t*-test).

The primary regulation of tomato fruit ripening involves ethylene and transcription factors, along with carotenoid accumulation and cell wall degradation [58]. To obtain further insights into the delayed ripening seen in *Slyabby2a* mutants, we compared the transcripts of previously reported ripening-related genes in both the WT and *Slyabby2a* fruit septum at the B stage. Quantitative RT-PCR analysis revealed a significant decrease in the transcripts of key genes involved in tomato fruit ripening, such as the ethylene biosynthesis rate-limiting gene *SlACS2* [59], ethylene receptor genes *SlETR3/NR* and *SlETR4* [60], and the “master ripening regulator” *SlRIN* [61], in the fruit septum of *Slyabby2a* mutants compared to the levels observed in the WT (Figure 7A–D). Additionally, the transcription levels of genes involved in carotenoid biosynthesis [62], including *SlPSY1*, *SlPDS*, *SlZDS*, and *SlLYCB* (Figure 7E–H), as well as cell wall metabolism genes [63], such as *SlPG*, *SlPE*, and *SlPL* (Figure 7I–K), were significantly downregulated in the fruit septum of *Slyabby2a* mutants. Furthermore, auxin plays a role in the unripe-to-ripe transition, and the small auxin-up RNA gene *SlSAUR69* positively regulates tomato fruit ripening [14]. To investigate whether the auxin signaling pathway is altered during fruit septum ripening in the *Slyabby2a* mutant, we examined the transcription levels of *SlSAUR69*. Quantitative RT-PCR analysis showed a significant downregulation of *SlSAUR69* expression in the fruit septum of the *Slyabby2a* mutants (Figure 7L).

Compared to the changes in septum ripening time, there was no significant difference in the ripening time of the fruit pericarp between the *Slyabby2a* mutants and the WT (Figure 6A). To explore whether *SlYABBY2a* affects the ripening process of the fruit pericarp at the molecular level, we selected genes with significant expression level differences in the septum and then quantitatively analyzed their expression levels in the fruit pericarp at stage B. The results showed no significant difference in the expression levels of key ripening-controlling genes, such as *SlACS2*, *SlETR3/NR*, and *SlRIN*, between the fruit pericarp of the *Slyabby2a* mutants and the WT (Figure S3). In contrast, the expression levels of *SlACS2*, *SlETR3*, and *SlRIN* in the fruit septum of the *Slyabby2a* mutants at the B + 2 stage remained significantly lower than those in the WT fruit septum (Figure S4). These results indicated that *SlYABBY2a* has a more significant impact on septum ripening than on pericarp ripening. Knocking out *SlYABBY2a* significantly reduces the expression levels of ripening-related genes in the fruit septum, ultimately leading to the delayed ripening phenotype observed in the *Slyabby2a* mutants.

### 2.7. SlYABBY2a Functions Downstream of MADS-Box Protein SlTAGL1

**Figure 8:**
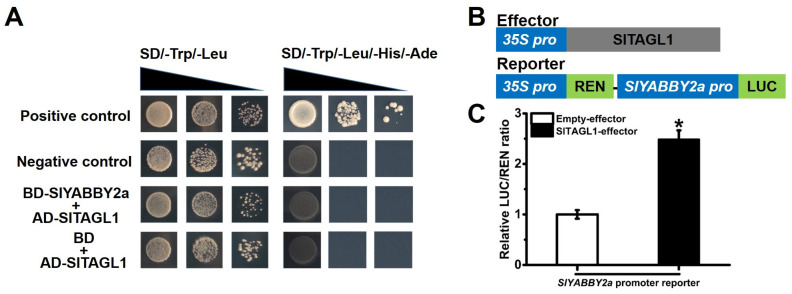
Interaction analyses between *SlYABBY2a* and *SlTAGL1*. (**A**) Transformation and protein-protein interaction in yeast. The related interaction confirmed by pGBKT7-53 and pGADT7-T served as a positive control, while pGBKT7-Lam and pGADT7-Tm served as negative controls. (**B**) Schematic diagram of constructs. (**C**) SlTAGL1 regulates the activity of the *SlYABBY2a* promoter as determined by dual-luciferase assays. Values are means ± SE (*n* ≥ 10) of three replicates. The asterisk indicates a significant difference determined by Student’s *t*-test (* *p* < 0.05).

Silencing or knocking out *SlTAGL1* leads to a phenotype of inward concavity in the tomato fruit septum [18,25], similar to what is observed in the *Slyabby2a* mutant (Figure 3). This phenomenon suggests a potential interaction between SlTAGL1 and *SlYABBY2a*. We first analyzed their interaction at the protein level using yeast two-hybrid (Y2H) technology. However, the results of the Y2H assay indicated that there is no direct interaction between the *SlYABBY2a* and SlTAGL1 (Figure 8A). Subsequently, we investigated the potential interaction at the transcriptional regulation level using a dual-luciferase reporter system, where SlTAGL1 was used as the effector and a promoter fragment of *SlYABBY2a* was used as the reporter (Figure 8B). Through transient expression in tobacco leaves and measurement of dual-luciferase activity, the results showed that SlTAGL1 can activate the promoter of *SlYABBY2a* and enhance the transcription of the downstream LUC gene (Figure 8C). These findings suggested that the transcription of *SlYABBY2a* is regulated by SlTAGL1, indicating that *SlYABBY2a* is a potential direct target of SlTAGL1.

## 3. Discussion

The *CRABS CLAW* (*CRC*) gene serves as the quintessential member of the plant-specific YABBY family, exerting a pivotal role in carpel development, and it is also indispensable for the formation of nectar-producing structures [64]. The precise expression pattern of *CRC* in carpel tissues [26] reflects the overarching function of YABBY genes in specifying the abaxial orientation of lateral organs in plants [32]. In the case of *Arabidopsis*, *crc-1* mutations result in impairments in carpel fusion and alterations in the overall size and shape of pistils [65]. Moreover, orthologues of CRABS CLAW (CRC) play a fundamental role in the determination of floral meristem (FM) and the formation of the gynoecium across angiosperms, both of which are pivotal developmental processes that ensure successful plant reproduction and crop yield [66]. Through a functional complementarity experiment, it was observed that the expression of *YAB2* under the control of the *CRC* promoter in *crc-1* mutants facilitated the complete fusion of developmentally compromised carpels, thereby suggesting a degree of functional similarity between *YAB2* and *CRC* [64]. Phylogenetic analysis conducted in this study revealed that the YABBY family can be classified into five distinct subfamilies, with two members (SlYABBY2a and SlYABBY2b) found in the tomato YAB2 subfamily and only one member (AtYAB2) present in *Arabidopsis* (Figure 1A). *SlYABBY2b* has long been regarded as a functional gene governing *fas*, a crucial locus implicated in the domestication of tomato fruit size [39]. Consequently, the YABBY family in tomatoes is also believed to be involved in regulating fruit shape [39]. However, recent investigations have demonstrated that the dominant gene at the *fas* locus is *CLV*, and its influence on fruit shape is mediated through the regulation of *WUS* transcription [41]. Although transgenic functional studies on *SlYABBY2b* suggest its involvement in the regulation of tomato plant height and fruit size [43], the mechanisms by which the tomato YABBY family TF governs fruit shape remain elusive.

The tomato fruit is a complex organ, consisting of various components from the inner to the outer layers. For instance, the internal structure includes the placenta, locular tissue, and septum [45]. Traditionally, only the pericarp has been utilized as a sample for assessing tomato fruit development and ripening, resulting in limited knowledge regarding the development and biological functions of the internal structures. Recent studies have indicated that an AGAMOUS MADS-box protein, namely SlMBP3, governs the pace of placenta liquefaction in tomatoes [46]. Further investigations have revealed that SlMBP3 acts as a critical regulator of locular tissue in tomato fruit, and deletion at the gene locus is responsible for the all-flesh trait [47]. These studies have shed light on the influence of internal structure development on fruit ripening and commercial characteristics. However, our understanding of the regulatory mechanisms governing septum development in tomato fruit still needs to be improved. Through transcriptome data analysis and qRT-PCR validation, we have identified a YABBY family gene, *SlYABBY2a*, which exhibits specific expression in the septum (Figure 3). Interestingly, the knockout of *SlYABBY2a* using CRISPR/Cas9 also affects the fusion degree of the carpels, further influencing fruit shape during fruit development and causing inward pericarp at the septum (Figure 4). Considering that the YABBY transcription factor CRC regulates proper auxin maxima, thereby influencing the initiation of carpel primordia and the termination of FM cell proliferation [49,50], we have examined the expression of genes involved in the auxin signaling pathway at the transcriptional level in the *Slyabby2a* mutant and WT septum. The results demonstrate a significant downregulation in the expression levels of genes related to auxin synthesis and transport, along with a significant upregulation in the expression levels of genes related to auxin inhibition in the *Slyabby2a* mutant compared to the WT (Figure 5). Moreover, alterations in the auxin signaling pathway have also affected the expression levels of cell cycle-related genes, such as *SlCycA3* and *SlCycD2* (Figure 5). Consistent with the phenotypes observed in the *Slyabby2a* mutant fruit, both silencing and knockout of the tomato *AGAMOUS-LIKE1* (*SlTAGL1*) result in an irregular fruit surface phenotype [18,25]. To investigate the potential correlation between these two similar phenotypes, we have explored the possible interaction between *SlYABBY2a* and *SlTAGL1* at the protein-protein interaction and transcriptional regulation levels. The research findings suggest that SlYABBY2a and SlTAGL1 do not directly interact in yeast, but in the dual-luciferase reporter system, SlTAGL1, can recognize the promoter of *SlYABBY2a* and enhance its transcription (Figure 8). In fact, *AtCRC* has been identified as a direct target of the MADS-box transcription factor AGAMOUS [51]. Therefore, these results indicated that *SlYABBY2a* is a potential direct target of SlTAGL1, further influencing carpel fusion and septum development by regulating the auxin signaling pathway. Subsequent investigations may involve overexpressing the *SlYABBY2a* gene in the WT or *Sltagl1* mutants to further validate the biological function of *SlYABBY2a*, as well as explore the interaction between *SlYABBY2a* and SlTAGL1.

Tomato, a typical climacteric fruit, has its ripening process strictly regulated by ethylene. Despite this control, the exact mechanisms behind the transition from immature to ripe stages are not fully understood. In the ripening process of climacteric fruits, there is a complex interaction between auxin and ethylene. For example, auxin plays a role in regulating ethylene biosynthesis and signaling genes in various fleshy fruits, like tomatoes and peaches [67,68]. Additionally, SlARF2 and SlARF4, which are auxin response factors, have been implicated in fruit ripening [69,70]. The importance of the interplay between ethylene and auxin is further evidenced by the influence of ethylene on the PIN1 auxin transporter and the need for high auxin levels for producing significant amounts of System 2 ethylene in peach [71]. Changing the levels of expression of auxin-responsive genes [72] can also influence the process of fruit ripening transition. The expression of *SlSAUR69* was increased in tomatoes, causing early ripening, while reducing it led to a delay in fruit ripening [14]. These findings strongly suggest that auxin plays a role in the transition to fruit ripening. In this study, we initially identified developmental defects in the septum of *Slyabby2a* mutant fruit, along with changes in the auxin signaling pathway characterized by a notable decrease in the expression levels of genes related to auxin synthesis (Figure 4 and Figure 5). As the fruit transitioned into the ripening stage, delayed ripening was observed in the septum of *Slyabby2a* mutant fruit, accompanied by a significant downregulation in the expression levels of ripening-related genes (Figure 6 and Figure 7), such as the ethylene biosynthetic gene *SlACS2* and other relevant genes. The expression of the auxin-responsive gene *SlSAUR69* in the septum of *Slyabby2a* fruit decreased by more than 80% compared to the WT counterpart (Figure 7L). Conversely, an analysis of key ripening-related gene expression in the fruit pericarp, including *SlACS2*, *SlETR3/NR*, and *RIN*, showed no notable differences between *Slyabby2a* and WT fruits (Figure S3). These findings suggest that the absence of *SlYABBY2a* specifically impacts septum ripening by modulating the auxin signaling pathway, subsequently affecting System 2 ethylene biosynthesis and downstream ethylene responses.

## 4. Materials and Methods

### 4.1. Plant Materials and Growth Conditions

In this study, *Solanum lycopersicum* cv. Ailsa Craig served as the genetic transformation materials, alongside wild-type (WT) controls. All tomato plants were cultivated in a controlled glass greenhouse, adhering to the following subsequent environmental parameters: 16 h of daylight maintained at 27 °C complemented by 8 h of nocturnal conditions at 19 °C whilst ensuring a relative humidity level of 70–80%. Materials from the roots, stems, leaves, flowers, and fruits of WT were collected for RNA extraction, facilitating the examination of *SlYABBY2a* expression levels across tissues and organs. Fully opened flowers were duly marked, and the stage of fruit development was recorded in days post anthesis (DPA). Fruits at the stage of 20 DPA were characterized as immature green (IMG), whereas those reaching 35 DPA were designated as mature green (MG). When the fruit surface visibly turned yellow, it was defined as the breaker stage (B), and the period of fruit ripening was distinguished by the number of days after the B stage, such as the B + 2 stage. Furthermore, tomato fruits underwent dissection into pericarp, septum, placenta, and columella for sampling, thereby facilitating RNA extraction and the subsequent analysis of *SlYABBY2a* expression in the internal structure of fruits.

### 4.2. Structure and Phylogenetic Analyses

The information regarding YABBY proteins in tomatoes, rice, and *Arabidopsis* referred to previous papers [27,28,29]. Subsequently, the conserved domains within each YABBY protein, along with their respective positions, were detected utilizing the ScanProsite tool (https://prosite.expasy.org/scanprosite/ (accessed on 10 October 2021)). Amino acid sequence alignment and phylogenetic analysis were conducted using MAFFT v7 [73] and MEGA11 v1 [74] software with default parameters, respectively. The accession numbers for the YABBY proteins utilized in this study are as follows: SlYABBY1a (NP_001353823.1), SlYABBY1b (NP_001353824.1), SlYABBY2a (NP_001353825.1), SlYABBY2b (NP_001234390.1), SlCRCa (XP_004228849.1), SlCRCb (XP_025886851.1), SlINO (XP_010321151.2), SlYABBY5a (NP_001353826.1), SlYABBY5b (XP_004251722.1), OsDL (NP_001389018.1), OsYAB1 (NP_001389953.1), OsYAB2 (NP_001389121.1), OsYAB3 (XP_015613818.1), OsYAB4 (NP_001403714.1), OsYAB5 (XP_015634596.1), OsYAB6 (NP_001067299.1), OsYAB7 (XP_015647119.1), AtFIL (NP_566037.1), AtYAB2 (NP_001077490.1), AtYAB3 (NP_567154.1), AtINO (NP_001320962.1), AtYAB5 (NP_850080.1), and AtCRC (NP_177078.1). In addition, the full-length amino acid sequences of the YABBY proteins are available in Table S1.

### 4.3. Expression Analysis of SlYABBY2a

To compare the expression levels of the 9 genes in the tomato YABBY family across different tissues and organs in tomatoes, gene expression heatmaps were generated using RNA-seq data from the Tomato Genome Consortium, 2012 [48]. The RPKM data, which represented gene expression levels, were normalized through log2 processing and visualized using the OmicStudio tool (https://www.omicstudio.cn/tool/ (accessed on 20 October 2021)). Furthermore, quantitative real-time PCR (qRT-PCR) technology was employed to validate the relative expression levels of *SlYABBY2a* in different tissues and organs. The qRT-PCR experiments were conducted with three biological replicates.

### 4.4. Subcellular Localization

To examine the subcellular localization of SlYABBY2a, a SlYABBY2a-YFP fusion protein and transient expression strategy was employed, with HY5-RFP fusion protein serving as a nuclear localization control signal. Firstly, the coding sequence of *SlYABBY2a*, without the stop codon, was cloned and digested with enzymes (*BamH*I and *Sac*I) before being inserted into the pHB-YFP vector, which had also been cleaved with enzymes (*BamH*I and *Sac*I), to construct the pHB-SlYABBY2a-YFP fusion vector. After transforming the plasmids expressing SlYABBY2a-YFP, YFP, and HY5-RFP into *Agrobacterium tumefaciens* GV3101-competent cells, positive monoclonal cells were cultured until reaching an optical density (OD_600_ = 1.0). Equal volumes of the GV3101 strains carrying pHB-SlYABBY2a-YFP and the control vector pHB-YFP were mixed with the GV3101 strain containing HY5-RFP, respectively, and the mixture was then infiltrated into 4-week-old tobacco leaves (*N*. *benthamiana*) using a needleless 1 mL syringe, followed by 48 h of cultivation. The infiltrated leaf sections were observed using a laser confocal microscope. Excitation wavelengths for GFP and RFP were set at 488 nm and 563 nm, respectively, and emission wavelengths for GFP and RFP were set at 507 nm and 582 nm, respectively. The cultivation of Agrobacterium and the preparation of the re-suspension solution were performed as previously described [75].

### 4.5. Gene Editing

The CRISPR-P tool (http://cbi.hzau.edu.cn/crispr/ (accessed on 1 November 2021)) was utilized to identify the knockout site on the *SlYABBY2a* gene. Combined with the usage method of the knockout vector pKSE-401 [76], primer pairs (forward: ATTGCAAACACGTTCCGAGGAAG; reverse: AAACCTTCCTCGGAACGTGTTTG) were designed and synthesized. Following denaturation and annealing, the primer pairs were inserted into the pKSE-401 vector and digested with *Bsa*I. The recombinant vector was transformed into *Agrobacterium tumefaciens* LBA4404 and utilized for a successful genetic transformation using cotyledons from the WT. Regenerated shoots were examined on selection medium with kanamycin (50 mg/mL), and regenerated plants were verified through PCR analysis of genomic DNA using primer pairs (*NPT*-II-F: TGTGCTCGACGTTGTCACTGAA and *NPT*-II-R: CACCATGATATTCGGCAAGCAG). Subsequently, the genomic target regions were cloned and sequenced to identify the genotype of the transgenic lines and whether potential off-target mutations occurred, as previously described [77]. In addition, Tables S2 and S3 list the specific primers used in this section, and Table S4 contains the detection results for potential off-target sites.

### 4.6. qRT-PCR

The samples stored at −80 °C were ground into a powder using liquid nitrogen. Following this, RNA isolation and reverse transcription reactions were carried out using TRIzol reagent (Invitrogen, Shanghai, China) and M-MLV reverse transcriptase (Promega, Beijing, China), respectively, according to standard procedures. The quality and quantity of RNA were evaluated as previously described [77]. The reverse-transcribed cDNA was then diluted with RNase/DNase-free water to three times its volume. For qRT-PCR analysis, a CFX96 Touch™ machine (Bio-Rad, Hercules, CA, USA) was utilized with a reaction system consisting of 5 µL of 2× GoTaq^®^qPCR Master Mix enzyme, 3.5 µL of nuclease-free water, 0.5 µL of primers, and 1 µL of diluted cDNA. The amplification conditions were as follows: an initial denaturation step at 95 °C for 2 min, followed by 40 amplification cycles (95 °C for 15 s and 60 °C for 40 s). The tomato *SlCAC* gene (Solyc08g006960) served as an internal reference gene [78], and the relative expression levels of the genes were determined using the 2^−∆∆CT^ method [79]. Each experiment included three biological replicates and three technical replicates.

### 4.7. Pigment Quantification and Histologic Analysis

The samples of different tomato fruits were ground into a powder using liquid nitrogen. Total carotenoid contents were extracted using a hexane/acetone solution, and the quantification was conducted by calculating the absorbance of samples as described in a previous study [80]. Lycopene extraction was achieved by employing a hexane/ethanol/acetone solution with 0.05% butylated hydroxytoluene (BHT), and the quantification was conducted by calculating the absorbance of samples as described in a previous study [81]. The paraffin sectioning of carpel tissue (one day after anthesis) using light microscopy was carried out according to previously established methods [82]. All measurements mentioned were performed in three independent experiments.

### 4.8. Yeast Two-Hybrid Assay

The yeast two-hybrid assay was conducted following the MATCHMAKER TM GAL4 Two-Hybrid System III protocol (Clontech, Palo Alto, CA, USA). In brief, the full-length open reading frame (ORF) of *SlYABBY2a* was inserted into the pGBKT7-BD vector and transformed into the Y2HGold yeast strain as bait strain. In contrast, the ORF of *SlTAGL1* was inserted into the pGADT7-AD vector as the prey. The recombinant bait strain containing pGBKT7-SlYABBY2a underwent a self-activation test as previously described [83]. After the self-activation test, the prey plasmids were transformed into the recombinant bait strain. The transformation and evaluation of protein–protein interaction were evaluated by screening on a DDO (SD medium without Trp and Leu) and QDO medium (SD medium without Trp, Leu, His, and Ade), respectively.

### 4.9. Dual-Luciferase Reporter Assay

The full-length ORF of *SlTAGL1* was cloned into the pGreen II 62-SK vector and then transformed into the *Agrobacterium tumefaciens* strain GV3101 as the effector. The promoter sequence of *SlYABBY2a* was also cloned and inserted into the pGreen II 0800-LUC vector, which was subsequently transformed into GV3101 as the reporter. Both the reporter and effector were co-transformed into tobacco leaves (*N*. *benthamiana*) for transient expression. The activities of firefly luciferase (LUC) and Renilla luciferase (REN) were measured according to a previously described protocol [84].

### 4.10. Primers and Accession Numbers

The qRT-PCR primers, along with the accession numbers for the target genes, were provided in Table S5. For vector construction, the primers were listed in Table S6, with partial primer sequences containing restriction enzyme sites.

### 4.11. Statistical Analysis

Statistical differences between the WT and each *Slyabby2a* mutant line, CR-1 and CR-2, were computed using Student’s *t*-test (* *p* < 0.05), while multiple comparisons were estimated using one-way ANOVA and Duncan’s multiple range test (*p* < 0.05), and the same lowercase letters indicate no significant differences.

## 5. Conclusions

**Figure 9:**
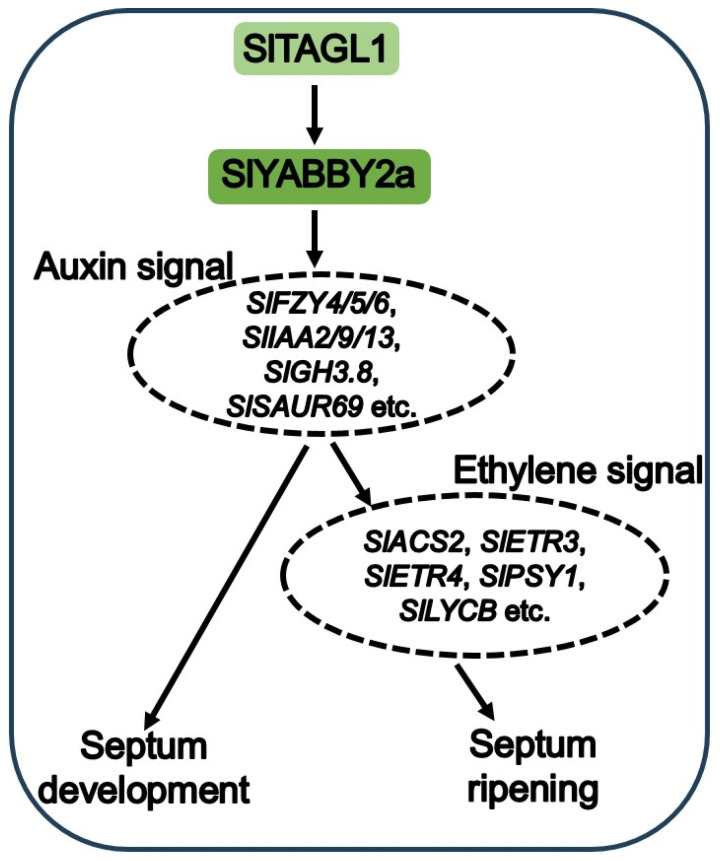
Proposed model depicting the regulation of SlYABBY2a and its role in controlling fruit septum development and ripening in tomatoes.

We have identified a novel tomato YABBY transcription factor, SlYABBY2a. Based on the results of knocking out *SlYABBY2a*, we hypothesize that SlYABBY2a positively regulates septum development and ripening by influencing the auxin and ethylene signaling pathways. A model has been proposed to illustrate the potential function of SlYABBY2a (Figure 9). In summary, our study contributes to the comprehension of fruit septum development and ripening and reveals that modifications in auxin signaling in the septum also impact the ethylene signaling pathway and the ripening process. This provides new evidence for the involvement of auxin in regulating the transition from unripe to ripe fruit.

## Data Availability

The data that support the findings of this study are available from the corresponding author upon reasonable request.

## Supplementary Materials

The following supporting information can be downloaded at https://www.mdpi.com/article/10.3390/ijms25105206/s1.
