# Supplementary material for: The YABBY Transcription Factor, SlYABBY2a, Positively Regulates Fruit Septum Development and Ripening in Tomatoes
Source: Int J Mol Sci. 2024 May 10;25(10):5206. doi: 10.3390/ijms25105206 (PMC11121019; doi:10.3390/ijms25105206)
Supplement: Supplementary file 1 [file ijms-25-05206-s001.zip › Supplementary Figures.pdf]

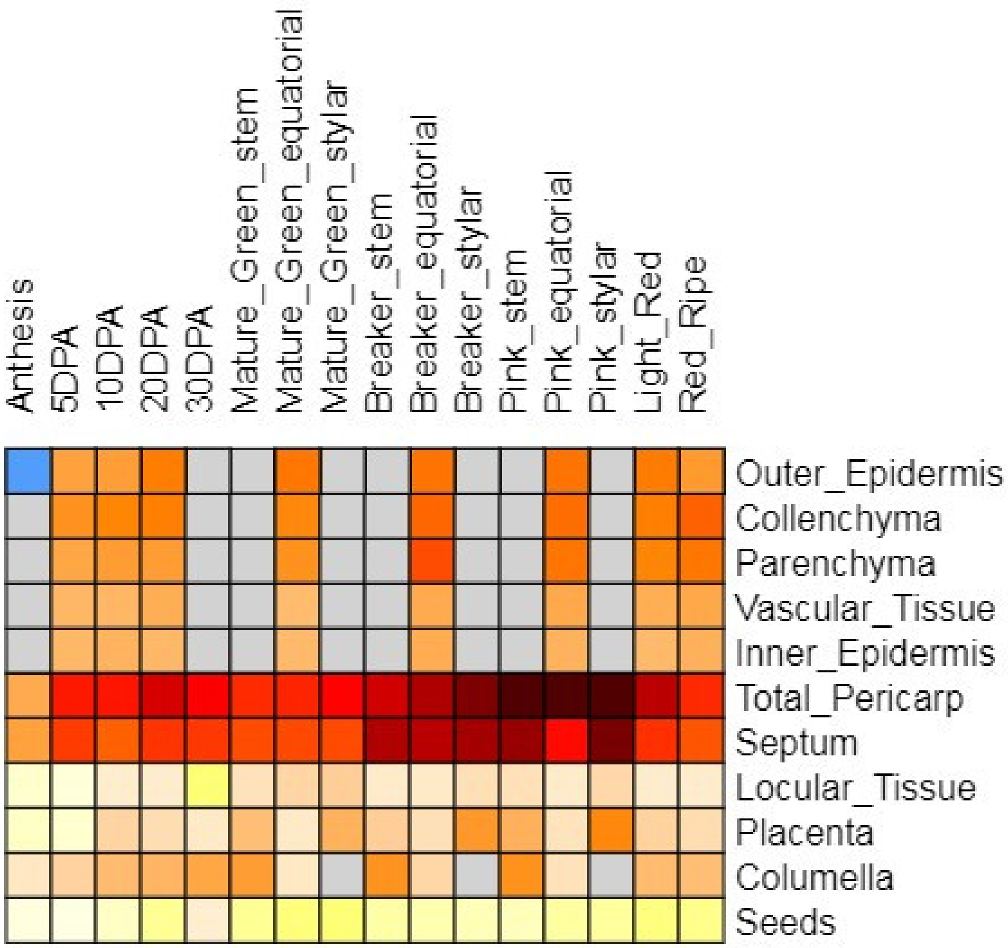

Figure S1. Expression analysis of *SlYABBY2a*. RNA-seq data and visualization were producted by Shinozaki et al., 2018 [45].

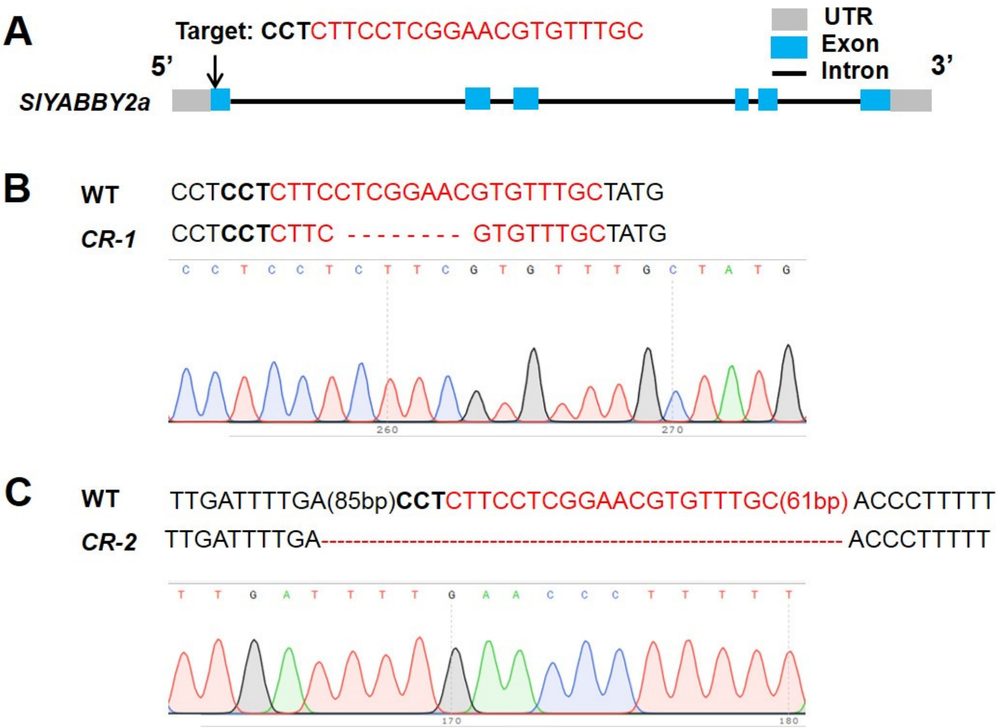

Figure S2. Knockout site and sequence alignment. (A) The gene knockout site was located in the first exon of *SlYABBY2a* and near the ATG start codon. **(B)** Genomic sequenes flanking the knockout site of wild-type (WT) and *CR-1* mutant. **(C)** Genomic sequenes flanking the knockout site of WT and *CR-2* mutant.

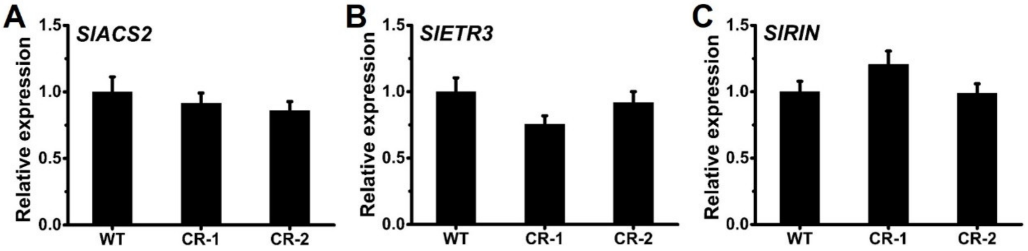

Figure S3. Transcripts quantification of fruit pericarp at the B stage from WT and *Slyabby2a* lines. (A-C) The relative expression of the ethylene biosynthesis gene *SlACS2* (A), the ethylene receptor gene *SlETR3* (B), and the master ripening regulator gene *SlRIN* (C). Each value represents the mean ± SE of three biological replicates.

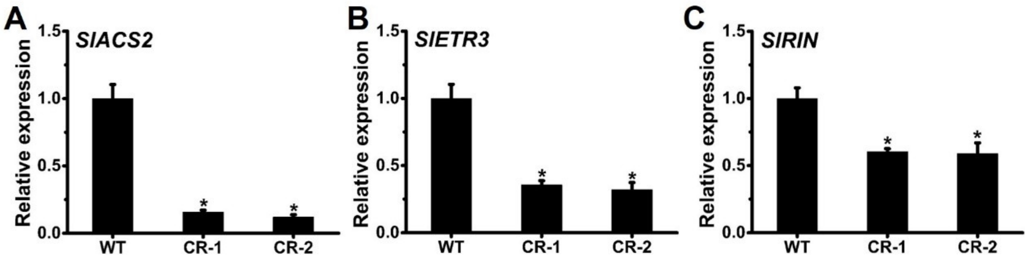

Figure S4. Transcripts quantification of fruit septum at the B+2 stage from WT and *Slyabby2a* lines. (A-C) The relative expression of the ethylene biosynthesis gene *SlACS2* (A), the ethylene receptor gene *SlETR3* (B), and the master ripening regulator gene *SlRIN* (C). Each value represents the mean ± SE of three biological replicates. Asterisks indicate significant differences between WT and each *Slyabby2a* mutant lines, CR-1 and CR-2 (* $p < 0.05$; Student's *t* test).
